# “Active Inference. The Free Energy Principle in Mind, Brain, and Behavior”

**DOI:** 10.17505/jpor.2026.29052

**Published:** 2026-03-26

**Authors:** Lars-Gunnar Lundh

**Affiliations:** Department of Psychology, Lund University, Sweden

**Keywords:** The free energy principle, Active Inference, generative models, model-world discrepancies, cognitive dissonance, self-discrepancies, placebo effects, mentalization-based treatment

## Abstract

This book is an introduction to the Free Energy Principle (FEP) and Active Inference. The FEP has been described as new paradigm with potential to unify the biological and cognitive sciences. According to the FEP, living organisms persist by minimizing their free energy (which can be variously translated as uncertainty, surprise, prediction error, or discrepancies between model and world). Active Inference means that we can resolve uncertainty (or discrepancies between model and world) in basically two ways: by perception (i.e., changing our mind to fit the world) and by action (i.e., changing the world to make it fit our preferences and beliefs). The FEP and Active Inference represent a conceptual framework that may have potential to contribute to a unification of psychological science.

The British neuroscientist Karl Friston is one of the most cited living scientists. During the last decades, he and his associates have written extensively on the *Free Energy Principle* and *Active Inference*. This work represents a new paradigm with ambitions to integrate concepts from theoretical physics, biology, neuroscience, psychology and other sciences. I first came across this new paradigm while reading Ginsburg and Jablonka’s (2019) work on the origins of consciousness, where they state the following:

Free energy minimization is the most general notion of how the organism can increase its adaptive fit to its environment: Darwinian selection can be seen as a special case of the free energy principle. (Ginsburg & Jablonka, 2019, p. 248)

As Kirchhoff et al. (2025) recently put it, the free energy principle

has been proposed as the basis for a grand unifying theory for the biological and cognitive sciences, identifying mathematical principles that can be applied to model a variety of biological systems at all scales of organization—from cells and multicellular organisms to cognitive processes such as perception, planning, action, and memory. (Kirchhoff et al., 2025, p. 640)

This naturally made me curious about this new paradigm and its potential implications for psychological science. In my attempts to familiarize myself with it, I found a book written by Thomas Parr, Giovanni Pezzulo, and Karl Friston with the title *Active Inference: The Free Energy Principle in Mind, Brain, and Behavior*.

## The Free Energy Principle

As described by Parr et al. (2022), the main question addressed by this theoretical approach is how “living organisms persist while engaging in adaptive exchanges with their environment” (p. 3). The basic answer they provide is that this is done by *minimizing their free energy*. This can be understood in the context of the second law of thermodynamics, according to which *entropy* (i.e., disorder) tends to increase over time. Living organisms, in contrast, show an opposite tendency to increased *order* (biological growth and development, increased knowledge about the world, etc.). To minimize free energy is to reduce entropy.

The second law of thermodynamics applies to closed systems, whereas living organisms are open systems in continuous interaction with their environment. Living organisms are constantly engaged in reciprocal interactions with their environment, by means of *action* and *perception*.

Living organisms can only maintain their bodily integrity by exerting adaptive *control* over the action-perception loop. This means acting to solicit sensory observations that either correspond to desired outcomes or goals (e.g., the sensations that accompany secure nutrients and shelter for simple organisms, or friends and jobs for more complex ones) or help in making sense of the world (e.g., informing the organism about its surroundings). (Parr et al., 2022, p. 3-4)

Basically, the free energy principle (FEP) involves the assumption that, to maintain its organization as an adaptive living system, the organism needs to *minimize the free energy* in its interactions with the environment. This leads to the question of *what* more precisely is minimized in this process.

### What is minimized?

What is minimized is described by the authors variously as “free energy”, “entropy”, “surprise”, “prediction error”, “uncertainty”, and “discrepancies between model and world”. Here are some examples of formulations:

this common objective has been described in various (informal and formal) ways, including the minimization of surprise, entropy, uncertainty, prediction error, or (variational) free energy. These terms are related to one another but sometimes their relations are not immediately clear, causing some confusion. (Parr et al., 2022, p. 25)

under some conditions, one can reduce variational free energy to other notions, such as the discrepancy between the generative model and the world, or the difference between what one expects and what one observes (i.e., a prediction error). (Parr et al., 2022, p. 25)

Surprise minimization permits living organisms to (temporarily) resist the second law of thermodynamics (Parr et al., 2022, p. 47)

As my interest here is in understanding the relevance of this paradigm specifically to psychological science, my aim was to understand *what is minimized specifically in psychological processes*, such as perception, action, emotion, thinking, communication, etc. Of the above-mentioned terms, some have an unmistakable psychological ring, such as “uncertainty” and “surprise”. But here it is important to not be misled by the everyday meaning of words like uncertainty and surprise. Intuitively, it seems that we have a natural willingness to reduce uncertainty. As to the minimization of surprise things are somewhat more complicated; although we generally don’t like *unpleasant* surprises, we may welcome *pleasant* surprises. What is at stake here, however, is not surprises as spoken of in our everyday language, but surprise as a term in mathematical information theory that quantifies the improbability of an outcome. To guard against misunderstandings, the term *surprisal* (rather than “surprise”) is often used in this mathematical context.

To me, it seems that a psychologically interesting term for what is minimized is *the discrepancy between model and world*.

both perception and action serve the very same objective. As a first approximation, this common objective of *perception and action* can be formulated as a minimization of the *discrepancy between the model and the world*. (Parr et al., 2022, p. 25)

As I understand it, the free energy principle as applied to psychological processes is assumed to take the form of a minimization of various kinds of experienced *discrepancies*. This seems to lie at the core of the theory of Active Inference.

## Active Inference

All living organisms are said to engage in active inference “in virtue of their existence” (Parr et al., 2022, p. viii). Active inference is described as a way of sampling the world for a purpose – to learn something new, to perceive more clearly, to understand better, or to decide what to do. In all these cases, the purpose is to resolve some kind of uncertainty about the world we live in:

Active Inference is a way of understanding sentient behavior. The very fact that you are reading these lines means that you are engaging in Active Inference—namely, actively sampling the world—in a particular way—because you believe you will learn something. You are palpating this page with your eyes simply because this is the kind of action that will resolve uncertainty about what you will see next and—indeed— what these words convey. (Parr et al., 2022, p. vii).

A basic assumption in Active Inference is that we can resolve uncertainty (i.e., minimize the discrepancy between our models of the world and the actual world) in basically two ways: by means of perception and by means of action:

one can minimize the discrepancy between a model and the world in two ways: by changing one’s mind to fit the world (perception) or by changing the world to fit the model (action). (Parr et al., 2022, p. 192)

By means of *perception* we can increase our clarity about the world around us. To take an example from the book, what first looked like an apple might, on closer look, turn out to be something quite different – maybe a frog. By changing our mind in this case, we reduce a discrepancy between our beliefs and the world. By means of *action*, we can change the world in another way - to make the *world* fit us better. For example, if it is too warm for our comfort, we may open a window to let in some cool air.

In both cases (perception and action, respectively), we reduce an experienced discrepancy. In the first case, we change our beliefs to make them *fit the world*. In the second case we change something in the world (the temperature in the room) to make it *fit our preferences*.

As I understand it, the latter kind of discrepancy must have arisen earlier in biological evolution. It is a kind of discrepancy which is intimately connected with the development of *life* as such. To survive, any living organism must maintain itself in a “comfort zone” that corresponds to a suitable set of preferred states, while avoiding other states that pose a threat to their survival:

to survive, any living organism has to maintain itself in a suitable set of preferred states, while avoiding other, dis-preferred states of the environment. For example, to survive, a fish has to stay in a comfort zone that corresponds to a small subset of all the possible states of the universe: it has to stay in water. Similarly, a human has to ensure that their internal states (e.g., physiological variables like body temperature and heart rate) always remain within acceptable ranges—otherwise they will die (Parr et al., 2022, p. 42)

As the authors point out, this comfort zone differs from one type of organism to another, and it is essential that the organism has the capacity to exert *active control* over the state that it is in. This control occurs at several different layers, from homeostatic physiological regulation, over psychological processes, to sociocultural practices:

This acceptable range or comfort zone stipulatively defines the characteristic states something has to be in to be that thing. Living organisms resolve this fundamental biological problem by exerting *active control* over their states (e.g., of body temperature) at many levels, which range from automatic regulatory mechanisms such as sweating (physiology) to cognitive mechanisms such as buying and consuming a drink (psychology) to cultural practices such as distributing air conditioning systems (social sciences). (Parr et al., 2022, p. 42)

The Active Inference paradigm is said to be equally appropriate for characterizing all kinds of living individuals, including simple organisms such as bacteria.

This renders Active Inference equally appropriate for characterizing simple creatures like bacteria that sense and seek nutrient gradients, complex creatures like us that pursue sophisticated goals and engage in rich cultural practices, or even different individuals—to the extent that one appropriately characterizes their respective generative models. (Parr et al., 2022, p. 194)

What characterizes different individuals is their different *generative models*:

while all creatures minimize their variational free energy, they behave in different, sometimes opposite ways because they are endowed with different generative models. Therefore, what distinguishes different (e.g., simpler from more complex) creatures is just their generative model. (Parr et al., 2022, p. 194)

If I understand this correctly, it means that the kind of models that developed first in evolution did not essentially involve any representations of *the world*, although they must have involved some kind of representation of *the organism’s preferred states*.

Furthermore, this means that generative models cannot be some kind of “copies” of the external world. They basically include *preferred* states (e.g., states within the comfort zone regarding temperature) that the individual needs to be in for their survival:

Importantly, the agent’s generative model cannot simply mimic external dynamics (otherwise the agent would simply follow external dissipative dynamics). Rather, the model must also specify the preferred conditions for the agent’s existence, or the regions of states that the agent has to visit to maintain its existence (Parr et al., 2022, p. 46)

In other theoretical approaches, this is commonly described in terms of *value* – each living organism is dependent on their environment for certain kinds of nutrients, safety from dangers, etc. These aspects of their environment are accordingly *valued* by the organism in the sense that they are actively approached (whether by simple tropisms, habits, goal-directed behaviours, or consciously planned actions). In the Active Inference paradigm, however, this is re-described in terms of the *belief* that *the preferred states are more likely to occur*. As Parr et al. put it, this means that the individual “has an implicit *optimism bias*” (p. 46). Here I have some difficulties following the authors’ reasoning. Should this really apply also to simple unicellular organisms such as bacteria? To me, beliefs about the likelihood of various events seem to be a much more advanced phenomenon that requires more complex representational capacities than those found in unicellular organisms.

An important point made by the authors here is that a good fit between model and world has both *descriptive* and *prescriptive* aspects:

A good fit indicates that the model successfully accounts for its sensations (this is the *descriptive* side of inference); at the same time, it realizes its preferred sensations, given that they are less surprising (this is the *prescriptive* side of the inference). (Parr et al., 2022, p. 47)

This formulation seems to recognize the importance of not reducing values (i.e., preferred states) to beliefs, as *prescriptions cannot be reduced to descriptions*.

Another term used here is “self-evidencing, defined as acting to achieve consistency between the internal model and the external world.

perception and action are both self-evidencing, in the sense that a creature can align what it expects, given its generative model, with what it senses either by changing its beliefs (about the presence of food) or by changing the world (soliciting food-related sensations). (Parr et al., 2022, p. 196)

All adaptive systems are said to engage in such self-evidencing (p. 47).

### Intentional action

Similar to perceptual processing, intentional action is also seen as a form of self-evidencing:

In Active Inference, action processing is analogous to perceptual processing, as both are guided by forward predictions—exteroceptive and proprioceptive, respectively. It is the (proprioceptive) prediction that “my hand grasps the cup” that induces a grasping movement.

In other words, action is seen to stem not from motor commands, but from anticipated consequences, consistent with the ideomotor theory of action and with Powers’ (1973) perceptual control theory, according to which action is controlled by perceptual states:

what is controlled is a perceptual state, not a motor output or action. For example, while driving, what we control—and keep stable over time in the face of disturbances—is our reference or desired velocity (e.g., 90 mph), as signaled by the speedometer, whereas the actions we select for this (e.g., accelerating or decelerating) are more variable and context dependent. For example, depending on the disturbance (e.g., wind, a steep road, or other cars), we would need to either accelerate or decelerate to maintain the reference velocity. (Parr et al., 2022, p. 203)

This kind of action control is seen to involve goals at different levels of hierarchical control, and motivational processes whereby more salient or urgent goals are prioritized (p. 203-204). Preferences obviously play a role here, but also beliefs (predictions) *that the goals will be reached*. This is seen as an optimistic bias

in the sense that the creature believes it will encounter preferred outcomes. It is this optimism that underwrites inferred plans that achieve desired outcomes in Active Inference; a failure of this sort of optimism may correspond to apathy (Parr et al., 2022, p. 207)

Intentional action among humans also includes the counterfactual capacity to consider alternative futures, in the form of *plans* and *policies*, and to “choose actions that look as if they are minimizing expected free energy” (p. 37). The notion of minimizing *expected* free energy is important, as it refers to choices that takes the *future* into account – in contrast to *variational* free energy which refers to choices in the present situation.

Variational free energy is at the core of Active Inference. It measures the fit between the internal generative model and (current and past) observations. By minimizing variational free energy, creatures maximize their model evidence. This ensures that the generative model becomes a good model of the environment and that the environment complies with the model. Expected free energy is a way to score alternative policies for planning. This is fundamentally *prospective*—it considers possible future observations—and *counterfactual*—the possible future observations are conditioned on the policies one could pursue. (Parr et al., 2022, p. 38)

### Communication

The authors illustrate how the Active Inference paradigm can be extended to communication between individuals with a simple example: birdsong. The basic idea here is that individuals with similar generative models predict the same kind of birdsong. If a bird does not hear the predicted birdsong, neither from itself nor from another bird, this becomes a prediction error and it starts singing:

This means that if a bird hears the song it is predicting, there is no need to generate it itself. However, if it predicts a song that is not heard, it must start singing to resolve any error. (Parr et al., 2022, p. 163)

This can develop into a kind of turn-taking between birds:

This dynamic becomes more interesting when there are two birds in play, with similarly structured generative models. As long as one bird is singing, the other does not need to, as there is no error to resolve. However, if one bird stops singing, the other needs to continue the same song. This leads to a form of turn taking, sometimes phrased as “singing from the same hymn sheet,” with each bird contributing sections of the same song… When there are two agents involved, this leads to an alternation between listening to the other and singing—a simple form of conversation. (Parr et al., 2022, p. 164-165)

The basic idea here is that individuals with *similar generative models* can synchronize their internal states in what may resemble “a primitive kind of theory of mind” (p. 163) – a generalized synchrony that may fail in humans suffering from neuropsychiatric syndromes such as autism (p. 165).

## The Dark Room Objection

An objection that has been raised against the FEP is that, if our guiding motive was to reduce uncertainty (surprisal), a perfect solution would be to stay in a dark room and never come out. Wouldn’t this eliminate all uncertainty and surprise in a most effective way? In his most recent book, Peter Godfrey-Smith (2024) reiterates this argument^1^:

If you really wanted to smooth the flow and reduce surprise, you would stay in a dark room and never come out, or come out as little as possible. That’s a choice that would *really* smooth things out. The limited appeal of this choice, and its dead-end status from a biological point of view, show that the point of perception and action is not just to reduce surprise. There’s more to life than that. (Godfrey-Smith, 2024, p. 79)

As far as I can see, however, this critique does not hit the FEP.

First of all, it is essential to note that the FEP involves the minimization of discrepancies between *model* and world. This means that “uncertainty” or “surprise” is always uncertainty/surprise *in relation to a specific model*. And because these models differ from one species to another, and from one individual to another (due to genetics, embodiment, experience, learning, etc.), *what is minimized needs to be assessed in each specific case*.

the free-energy principle will need to be unpacked carefully in each sphere of its application. This is the real challenge ahead. (Friston et al., 2012, p. 6)

In view of our specific human models of the world, it is highly unlikely that we would prefer to stay in a dark room:

we act to reduce expected surprise or, more simply, *resolve uncertainty*. So what’s the first thing that we would do on entering a dark room—we would turn on the lights. Why? Because this action has epistemic affordance; in other words, it resolves uncertainty (expected free energy). (Friston et al., 2018, p. 26)

Turning on the light makes us able to *see* – it thereby affords visual exploration of the room we are in, to resolve uncertainty about it. Our generative models, as discussed above, involve not only predictions but also *preferences* – for example, preferences for light versus darkness, and a motivation to engage in visual exploration of the environment.

Still, there are other organisms that seem to prefer at least something similar to a dark room – a cave:

Interestingly, Dark-Room agents do exist: Troglophiles have evolved to model and navigate environments like caves (Friston et al., 2012, p. 2)

Our specifically *human* generative models are the result of a long biological evolution, and they involve a preference for light against darkness. For us, darkness involves considerable uncertainty, which we strive to minimize. But things are different for other organisms such as troglophiles that have evolved models to predict and inhabit other kinds of environments

According to Friston et al. (2012), surprise is minimized *over multiple scales*. As I understand it, this involves minimization of uncertainty over at least three different scales:

1. the *development* of models during the *evolution* of the human species that made us able to minimize uncertainty and thereby adapt to the environment in our specific human ways;
2. the *shaping* of these models by *learning* experiences that make individuals able to minimize uncertainty in more specific ways during their life-span; and
3. the *choice* among existing models in the *interpretation* of specific situations that make us able to minimize uncertainty about the meaning of specific objects, events, and interactions in the here-and-now (the action-perception loop).

Moreover, the action-perception loop is not only a matter of interpreting *actual* situations but also involves plans, projects, and policies *for the future*. In the latter case, what is primarily minimized is not variational free energy in the present but rather *expected* free energy in the future. In fact, we are willing to accept *short-term increases* in uncertainty to resolve uncertainty *in a longer time-perspective*:

we usually need to accept some short-term increase of entropy or surprise (e.g., when we build something new or shift social stances) to ensure their long-term decrease. This helps us understand how the basic requirement for surprise minimization is not at odds with but rather promotes the epistemic imperatives and novelty-seeking, curious, and exploratory behavior that we recognize as central to many species. (Parr et al., 2022, p. 60)

To engage in exploration means to expose oneself to uncertainty *in order to resolve it*. The preference for knowledge, understanding, and clarity is an essential part of our human-specific generative models, and this clearly motivates us to move beyond the short-term resolution of prediction errors.

The dark room objection obviously does not affect the FEP. At the same time, however, all this points to the need for a much more detailed understanding of *the nature of generative models*.

## The Nature of Generative Models

During the history of psychological science, many researchers have recognized the need for a way of conceptualizing the structures in the mind/brain that have develop as a result of evolution and are then shaped by individual experience, and that can explain the variation in how different individuals perceive the world and act in relation to it. In FEP and Active Inference, the term used is “generative models”. The perhaps most common term for these kinds of structures that has been used historically in the psychological literature is “schema” or “schemata” (e.g., Bartlett, 1932; Neisser, 1976; Piaget, 1967); still that term has never become anything more than a “placeholder” for more precise concepts that need to be developed.

The theory of the mind as a system of meaning structures (Lundh, 1983, 1995) was an attempt to develop a more specific theory about these structures, seen as involving three dimensions of meaning; (1) *extension* (e.g., perceptual categorization and differentiation), (2) *intension* (e.g., beliefs, expectation), and (3) *value* (e.g., preferences, motives). The notion of generative models in the FEP/Active Inference paradigm captures two of these dimensions: beliefs and preferences. Little is said, however, about the categorization/differentiation dimension. According to Edelman (1992), perceptual categorization is one of the most fundamental processes of the vertebrate nervous system.

Another key aspect of our generative models is that they include models of ourselves and of other individuals:

a key component of any model (especially for social agents) will be a model of conspecifics. In other words, my model of the world will include a model of you, which will include your model of me and so on (Friston et al., 2012, p. 4).

This means that the generative models must capture our ability to model *other persons* in terms of their beliefs, desires, intentions, emotions, thoughts, etc. The development of these models has been empirically studied in research on the child’s “theory of mind” (e.g., Apperly & Butterfill, 2009). This research is mostly focused on the child’s *beliefs* about others’ minds. However, these models also need to integrate an individual’s *preferences* for other individuals, and our ability to *categorize* and *differentiate* between our own and others’ beliefs/desires/intentions/emotions/thoughts.

I guess a refined understanding of the nature of our generative models (schemata, meaning structures) may open up for an extended application of the FEP/Active Inference paradigm to other branches of psychological science. As Parr et al. (2022) put it, this paradigm

offers a first principle account of the ways in which organisms solve their adaptive problems. The normative approach pursued in this book assumes that it is possible to start from the principle of variational free energy minimization and derive implications about specific cognitive processes, such as perception, action selection, attention and emotion regulation, and their neuronal underpinnings. (Parr et al., 2022, p. 195)

In other words, their claim is that Active Inference can serve as an integrative paradigm for *cognitive* science. But what about the whole of *psychological* science? This takes the question to processes at a higher level of complexity. Does FEP and Active Inference have potential to contribute to the development of an integrative psychological science?

More complex psychological processes can be either intrapersonal or interpersonal. I will end this review with a little speculation about the integrative potential of the FEP and Active Inference when it comes to the conceptualization of some more complex psychological processes.

## Some Speculations

There are several theoretical approaches in psychological science that focus on *intrapersonal* model discrepancies. An early illustration that seems quite compatible with FEP is Festinger’s (1957) cognitive dissonance theory, according to which people experience mental discomfort when they hold contradictory beliefs, values, or attitudes – a kind of discomfort which is assumed to motivate people either to change their behavior or beliefs to restore consistency and reduce discomfort.

Another influential theory along partly similar lines is Higgins’ (1987) self-discrepancy theory, according to which different kinds of *self-discrepancies* cause different kinds of discomfort. Higgins differentiates between *actual self* (the attributes one believes that one has), *ideal self* (the attributes that one would like to have), and *ought self* (the attributes that one believes one ought to have). Among other things, Higgins differentiates between *actual-ideal discrepanci*es and *actual-ought discrepancies*. It is probably quite possible to integrate this kind of theory with an FEP/Active Inference perspective. In such a perspective, self-discrepancies might be minimized either (1) by changing one’s *beliefs* about oneself (either about one’s actual self, or about how one ought to be); (2) by changing one’s *preferences* about how one would like to be (i.e., the ideal self); or (3) by changing one’s way of *being in the world*. All kinds of “reality testing” (e.g., as carried out in cognitive therapy) would belong to the first category, whereas all attempts at self-improvement, “self-realization”, personal development, etc., would belong to the third category.

As to *interpersonal* model discrepancies, they seem to lie further away from the existing FEP literature. But I guess the FEP paradigm could be used also to conceptualize such discrepancies. This is a huge area, which includes not only clashes between different religious and political beliefs and between competing theories in science but also, at a more modest level, the divergencies in personal beliefs that turn up between people in all interpersonal communication. From the perspective of Active Inference, it seems that such discrepancies can be reduced either (1) if person A changes their beliefs to make these *conform* to those of person B, or (2) if person A *persuades* person B to change their beliefs in a way that makes these more similar to those of person A.

This might, for example, offer a way of conceptualizing placebo effects in medicine as well as certain kinds of “common factors” in psychological treatment. Placebo effects may be understood as deriving at least partly from a doctor’s persuasive communication of positive beliefs about the effects of a treatment, where patients are led to change their beliefs to fit those of the doctor:

an important part of the placebo effect is due to the development of placebo beliefs (beliefs of the form ”This treatment is going to cure me”), which may counteract the kind of cognitions that produce anxiety and depression; placebo beliefs produce emotional responses (hope, calm, etc.), which are antagonistic to depression and anxiety. (Lundh, 1987, p. 128)

In psychotherapy research, a debate has been going on about the importance of placebo-like factors referred to as “common factors” (for a theoretical review of this debate, see Lundh, 2014). One of the main proponents of the common factors view, Jerome Frank, has argued that most of psychotherapy is to be seen as “a form of rhetoric best studied hermeneutically” (Frank & Frank, 1991, p. 53), rather than as an applied behavioral science. Although there *is* evidence for specific effects of at least some psychological treatment methods (i.e., effects that cannot be reduced to “common factors”), a large part of the effects obtained in present forms of psychotherapy can probably be best understood in terms of the communication between therapist and patient, where one basic process is that patients gradually change their beliefs and attitudes to converge with those of the therapist.

Here, however, it is also interesting to note that some therapeutic techniques are *explorative* in the sense that the therapist asks open questions both to explore the patient’s experiences and to explore possibilities for change in the patient’s life, without any explicit attempt at persuasion. Some therapists, for example, prescribe a “not-knowing stance”. For example, a basic idea in Mentalization-Based Treatment (MBT; Bateman & Fonagy, 2004) is that there will inevitably occur “mismatches” in a therapist’s understanding of the patient’s experiences. I guess such mismatches can be conceptualized as interpersonal discrepancies between two models (the therapist’s and the patient’s). If the therapist takes a “not-knowing stance” and explores these mismatches together with the patient, this may help to resolve these mismatches. As Bateman and Fonagy (2004) puts it, “the therapist has to be able to examine his own internal states and be able to show that they can change according to further understanding of the patient’s state” (p. 210). In other words, *the therapist’s* beliefs are changed during the exploration of the patient’s experiences, and *the patient’s perception* of this change in the therapist is assumed to have therapeutic effects in turn. This might perhaps be seen as yet another illustration of how it is possible for one person (in this case the therapist) to expose themselves to uncertainty (a “not-knowing stance”) in order to arrive at a reduction of uncertainty later on (minimization of *expected* free energy) – in this case in both therapist and patient.

One thing that fascinates me about the new perspective provided by the FEP is that it suggests that basic human strivings not only for health and survival but also for well-being, knowledge, understanding and clarity may be variations of a fundamental principle that can be traced back to the very origins of life, and even further back in time, and that may possibly help to explain why the world has developed in the way it has, despite the second law of thermodynamics.


*Lars-Gunnar Lundh*


## Footnotes

1 Godfrey-Smith explicitly directs this argument against another theory – predictive processing theory – which shares important elements with FEP and Active Inference.

## Open access

This article is distributed under the terms of the Creative Commons Attribution 4.0 International License (http://creativecommons.org/licenses/by/4.0/), which permits unrestricted use, distribution, and reproduction in any medium, provided you give appropriate credit to the original author(s) and the source, provide a link to the Creative Commons license, and indicate if changes were made.
